# The origins of the treatment of uncertainty in clinical medicine – Part 2: the emergence of probability theory and its limitations

**DOI:** 10.1177/0141076820928901

**Published:** 2020-06-10

**Authors:** Robert AJ Matthews

**Affiliations:** Department of Mathematics, Aston University, Birmingham B4 7ET, UK

## Introduction

[I]t is a very certain truth that, when it is not in our power to discern the truest opinions, we ought to follow the most probable.^1^

Physicians since at least the time of Hippocrates have recognised that diagnosis, treatment and outcome are subject to uncertainty. The use of probabilistic reasoning to address this reality has similarly ancient roots. Yet in classical times, such reasoning was essentially restricted to ‘balance of probability’ arguments based on simple comparisons of proportions.^2^ As such, it failed to deal with the full consequences of uncertainty in decision-making. These only became apparent after the emergence of the mathematical theory of probability in the mid-17th century. This allowed the formal treatment of issues which had long been recognised in qualitative terms, such as the role of sample size in determining the reliability of ‘balance of probability’ arguments.

In the second part of this history, I examine how the theory of probability, initially seen by some as a panacea for the treatment of uncertainty in medicine, quickly sparked bitter disputes about its implications and relevance which continue to this day.

## The quantification of the play of chance

A critical turning point in the quest for certainty in clinical medicine was reached during the Renaissance: a recognition of the role of quantitative measurement. The pivotal figure here is the Italian physician Santorio Santorio (1561–1636). After studies in both medicine and mathematics at the University of Padua, Santorio began a life-long quest to bring more certainty to diagnosis and treatment. He noted that while Galen accepted that effective treatment required knowledge of both the type of disease and its severity, he had failed to specify how the latter could be anything other than guesswork.^3^ Santorio believed the way forward lay in the design and manufacture of precision instruments, combined with repeated and controlled observation. To this end, he constructed instruments capable of quantifying medically relevant factors including weight, temperature and pulse rate. For example, his development of the thermometer allowed medicine to move beyond Galen's simplistic diagnostic concepts of hot/cold and dry/moist towards quantitative measurement of a condition such as fever.

Santorio's remarkable (and remarkably little-known) role in the quest for certainty in medicine seems at least partly motivated by a desire to show that the discipline could achieve the Aristotelian status of a ‘true’ science. The quantification of measurement is, however, insufficient for achieving such status, as uncertainty still accompanies such measurement. Recognition of this in a clinical context can be found in Ibn Sina's seven rules (discussed in Part 1 of this history^2^), which in essence address various sources of uncertainty that can undermine the value of quantitative evidence, specifically bias (Rules 1, 2 and 7) and the play of chance (Rule 6).

Of these sources of uncertainty, the role of chance was the first to be formally addressed using mathematics. This followed the emergence of the theory of probability. The connection between games of chance and the acquisition of uncertain knowledge was recognised over 2000 years ago by the Roman orator and philosopher Cicero (106–43 BCE). He also noted that while uncertain knowledge is not completely reliable, it can still be useful, citing medicine as a field where this is true (Franklin,^4^ p.164). The first quantitative application of probability has been ascribed to Al-Kindi, yet this was in the field of cryptanalysis (‘code-breaking’).^5,6^ With its reliance on the analysis of sufficiently many intercepted messages to translate ciphertext into plaintext, this work led Al-Kindi to note the need for adequate sample sizes as a basis for reliable inferences. Perhaps surprisingly, however, there is no evidence that Al-Kindi explored the implications of this in relation to medicine, despite his work on drugs.^7^

Applications to medicine were certainly not the immediate concern of the 17th century European mathematicians who laid the foundations to probability theory. Their focus was the solution of questions arising in games of chance, exercises in aleatory probability which were regarded as frivolous even at the time.

What may have been the first such application concerned questions in physiology. Archibald Pitcairne (1652–1713), a Scottish physician and professor of medicine at the universities of Edinburgh and Leyden, had been inspired by the success of William Harvey and Isaac Newton in using quantitative methods to reveal truths about the natural world, and became convinced medicine could benefit from a similar approach.^8^ From 1688 until the mid-1690s, Pitcairne became a vocal advocate for mathematics as a golden road to certainty in medical matters. In 1693, he investigated questions concerning secretory processes using arguments published decades earlier in the first treatise on mathematical probability – *De ratiociniis in ludo aleae* (‘On reasoning in games of chance’; 1657) by the Dutch mathematician Christian Huygens. Especially notable is Pitcairne's use of quantitative inference to turn Santorio's instrumental measurements of body weight into guidance on how best to cure fevers.^8^ Given the belief that any such cure requires the expulsion of whatever had caused the fever, Pitcairne argued that the probability of a cure will be in proportion to the rate at which expulsion occurs. Drawing on Santorio's determination that the rates of bodily excretion through stool, urine and the pores of the skin are in the ratio 1:4:10, Pitcairne concluded that fevers are 10 times more likely to be cured by encouraging perspiration than by purgatives. However, this attempt to put medicine on a ‘Newtonian’ basis provoked scorn from some of his influential contemporaries. This was partly the result of somewhat esoteric religious and political tensions then rife in Scotland.^9,^^10^ Of more contemporary relevance, however, was criticism of Pitcairne's view that the human body can be modelled as some kind of machine, akin to the idea of ‘celestial clockwork’ underpinning Newton's conception of the universe.

Pitcairne's most vociferous critic was fellow Scottish physician Edward Eizat, who declared in an anonymous pamphlet (*Apollo Mathematicus* 1695)^11^:

Did ever any thing more wild or extravagant enter into the Mind of Man, than to imagine that this speculative Science, that goes all by Demonstration [ie mathematical proof], shall be of use in a practical Art founded on Experience? (p.18, quoted in Stigler^8^)

Behind the vituperation were arguments that were themselves based on sophisticated mathematics. Of particular relevance to the present review is Eizat's rejoinder to Pitcairne's claim that ‘ … nothing is infallibly certain, but a [mathematical] Demonstration’ (Friesen,^9^ p.172). As evidence, Pitcairne cited the most celebrated fact of plane geometry, Pythagoras’ Theorem. Eizat countered that even the most basic proven facts about triangles can be no more certain than a historical truth, as both are based on assumptions capable of challenge. To those unfamiliar with the existence of non-Euclidean geometry, this may seem an absurd statement; it has classical roots, however, and is mathematically well founded.^a^ Yet despite attacking Pitcairne's confidence in the power of mathematics, Eizat stressed he did not reject the use of mathematics in medicine out of hand. He was, he insisted, concerned with the potential for abuse of its powers through cavalier application – another strikingly contemporary concern.

This did nothing to appease Pitcairne and his supporters, one of whom authored an equally vituperative response later that year.^12^ It dismissed Eizat (though without naming him) as variously among those ‘[W]ho cry out upon the use of Mathematics in Physick [medicine]’ despite being ‘intirely ignorant of Mathematicks’, preferring instead to put their trust in experience. This attack on Eizat's mathematical knowledge was, as we have seen, wholly misplaced and in places highlights the author's own ignorance. Yet as with Eizat's pamphlet, the polemic makes some valid criticisms, notably the threat posed to observations by what is now called bias. This did not prevent both Hepburn and Pitcairne being censured by the Royal College of Physicians in Edinburgh for the ‘calumnious, scandalous, false and arrogant paper’, a judgement based as much on political as intellectual grounds. Of more historical importance is the fact that while Pitcairne's work influenced some notable fellow physicians, among them Herman Boerhaave, George Cheyne and Richard Mead, it failed to inspire the more widespread use of mathematics to reduce uncertainty in medical science. This was doubtless partly due to natural resentment on the part of physicians to the encroachment of unfamiliar techniques on their area of expertise. However, as Eizat's polemic makes clear, there was also well-placed suspicion that such techniques may be used inappropriately and divorced from qualitative medical knowledge. In light of what we now know,^13^ Pitcairne's analysis of how best to ‘treat’ fever appears to be a case in point, having reached the right conclusion on the basis of flawed logic and evidence.

## The emergence of modern methods

It took a mathematician of far greater gifts than those of Pitcairne and his acolytes to take the first steps towards using mathematics to treat uncertainty in medicine. Ironically, the outcome was an insight into the *limitations* of mathematics in this role, and one which influences clinical research to this day.

Born in Switzerland, Jacob Bernoulli (1655–1705) was the eldest member of the most famous mathematical family in history. In his 20s, after some work on games of chance, he turned his attention to the emerging theory of probability. During the winter of 1685–1686, he began to explore the re-interpretation of probability as more than simply the proportion of successes in a series of trials but as a means of gauging the validity of a model of events under study. That in turn led Bernoulli to investigate the probability of applying the theory to more general questions subject to uncertainty, among them the effectiveness of medicines.^14^ Bernoulli wanted to extend the theory developed for games of chance to such non-trivial matters to allow, for example, the description of a treatment as ‘probably’ effective if patients had a better than 50% chance of recovery. However, to do so he had to confront the problem that, in contrast to a pack of cards, we do not know the nature of the full ‘deck’ of patients from which those treated are to be ‘drawn’ with equal probability. As such, the true probability of the effectiveness of the treatment can only be estimated from the proportions observed from a given (random) sample of patients.

Bernoulli set out to discover the relationship between this estimate and the true probability and found it during the winter of 1689–1690 through a six-page proof of what has become known as the Law of Large Numbers. In essence, it shows that as the size of the sample increases, the risk that the proportion of events observed is wildly different from their true probability becomes ever smaller. As such, Bernoulli's theorem may seem merely a rigorous demonstration of what Bernoulli himself insisted was known even to ‘the stupidest man’: that the inclusion of more data reduces uncertainty.

Bernoulli was clearly ambitious for what he called the *Theorema Aureum* (‘Golden Theorem’), declaring: ‘I prize this discovery more highly than if I had given the very quadrature of the circle [a mathematical problem unresolved for another two centuries]: for even if this were discovered it would be of little use’. He intended making it the culmination of his planned *magnum opus* on probabilistic reasoning: *Ars Conjectandi* (‘The Art of Conjecturing’), the final part of which was to be devoted to real-life problems beyond games of chance. As a demonstration of its power, he applied his theorem to the venerable problem of determining the amount of data required to provide compelling evidence. Bernoulli prefaced his demonstration by giving an example of the kind of problem he had in mind: for estimating the chances of a man dying within the next 10 years, given that observation had shown that out of 300 men ‘of the same age and temperament’, 200 had died. According to Bernoulli ‘we may conclude sufficiently safely’ that the man faces 2 to 1 odds of dying within 10 years. Bernoulli then set about showing how his theorem could answer the question that had defied solution for millennia: how big a sample is required to constrain these odds to *any* degree of ‘safety’.

Before describing the startling outcome, it is worth noting that in his mortality example, Bernoulli reveals his belief that a cohort of just a few hundred would suffice to give an acceptably ‘safe’ estimate. As we shall see, Bernoulli may thus be regarded to be the first to perform a sample size calculation, and also to have had a wholly unrealistic expectation of its outcome.

To demonstrate the practical uses of his theorem, he asked the reader to imagine an urn containing 3000 white pebbles and 2000 black ones. The true probability of picking a white stone from the (presumably thoroughly mixed) urn is thus 60%. But, asked Bernoulli, how many stones would have to be sampled with replacement in order to be 99.9% certain that the probability has been established to within 2% of its true value? After some rather abstruse calculation, Bernoulli extracted an answer: a ‘safe’ estimate would require the examination of 25,500 stones.

This is, of course, a ludicrous outcome; simply tipping the 5000 stones out of the urn and counting them would be both faster and give the exact answer. Bernoulli's reaction to his finding is unclear; what is clear is that after adding an account of the calculations to the manuscript of *Ars Conjectandi* he ceased work on his putative *magnum opus*. In correspondence with the German mathematician Gottfried Leibniz, Bernoulli said poor health and ‘innate lethargy’ militated against its completion (Mattmüller,^14^ p.285). There are, however, also hints of disappointment that the resulting sample size was so much larger than expected or – more seriously – available for the applications he envisaged.

*Ars Conjectandi* remained unpublished until 1713, eight years after Bernoulli's death. It is now widely viewed as the seminal text in the application of probability to non-trivial problems.^15^ However, its *Theorema Aureum* is also the first demonstration of a key theme in the quest for certainty in clinical medicine: the disjunction between what practitioners would like to be possible, and practical reality. This disjunction can be seen in Bernoulli's bizarre failure to relax the standards of certainty he sought from his theorem. In modern terminology, he was asking for the sample size needed to achieve precision in the proportion of ±2% at the 99.9% confidence level. Intuition suggests – and as we shall shortly see, modern theory confirms – that being more flexible considerably reduces the sample size. Yet Bernoulli, seemingly fixated on establishing the proportion with ‘*moralis certitudo*’ [moral certainty] failed to investigate the effect of relaxing his demands; indeed, he chose instead to state the even more ludicrous sample sizes resulting from even more demanding confidence levels.

While Bernoulli's reasons for failing to publish his findings remain unclear, it may be that his disillusionment led him (rightly) to question the technical basis of his calculations. The requisite technical advances were introduced 25 years after his death by the French mathematician Abraham De Moivre. Investigating their impact on Bernoulli's estimate, Pearson^16^ showed they result in the required sample size plunging by almost 75% from 25,500 to the far more reasonable (and practicable) figure of around 6500. Indeed, so great is the improvement achieved by De Moivre's refinements that Pearson argued that Bernoulli's role in applying probability theory to practical matters ‘has not the importance which has often been attributed to it’. Certainly, De Moivre deserves credit for making Bernoulli's theorem of practical value. His formulation also reveals the impact of demanding such high levels of confidence or precision from sampling. Reducing the confidence level from Bernoulli's ‘moral certitude’ of 99.9% to today's conventional confidence level of 95% cuts the required sample size by almost two-thirds, to around 2300, while additionally relaxing Bernoulli's level of precision to ±3% (giving the standard of evidence now widely adopted by opinion pollsters) the figure drops again to around 1000.

Bernoulli's work on the Law of Large Numbers thus clearly constitutes a major conceptual advance in the application of mathematics to practical issues, including clinical medicine. However, his goal of making the theory of probability of practical value eluded him because he lacked the necessary mathematics and demanded a very stringent level of evidence. Whether he would have been willing to accept the less demanding standards of evidence used in modern research is unclear.

## Conclusion

With its central aim of identifying effective treatments for potentially life-threatening conditions, clinical medicine has an especially pressing need to minimise uncertainty. Over the last 2500 years, this has led to the adoption of a range of approaches from simple experience through logic to the use of probability theory. All have been the subject of often vociferous debate which continues to this day. The emergence of the theory of probability in the mid-17th century set the stage for today's focus on statistical methods as the principal quantitative means of addressing uncertainty in clinical medicine. This did not happen overnight, however; there were qualms about the relevance and reliability of these methods from the outset. Even as the theory became more sophisticated during the 19th century, its implications for the numbers of patients required to attain ‘compelling’ evidence, and the relevance of that evidence to individual patients, provoked bitter debate.^17,18^ While the means of reducing uncertainty in clinical medicine have evolved over the millennia, this debate remains as current and crucial as ever.

## Note

Eizat makes reference to Euclid's classical proof that the internal angles of a triangle sum to two right angles (*Elements* Book 1, Proposition 32). As a demonstration of mathematical uncertainty, this may seem ill-conceived. However, as Eizat appears to have known, Aristotle noted the existence of certain types of triangle where Euclid's proof may not hold.^19^ During the 19th century, this was formally traced to a violation of Euclid's fifth postulate – that parallel lines only meet at infinity. It is somewhat ironic, therefore, that Hepburn sought to attack Eizat on the grounds that he ‘does not understand’ Pythagoras’ Theorem, as that is another of Euclid's proposition whose validity rests on the fifth postulate, and thus supports Eizat's contention that not even mathematics offers absolute certainty.

## Declarations

### Guarantor

RAJM.

### Provenance

Invited article from the James Lind Library.
